# Circadian rhythm disrupting behaviours and cancer outcomes in breast cancer survivors: a systematic review

**DOI:** 10.1007/s10549-022-06792-0

**Published:** 2022-11-23

**Authors:** Kelly D’cunha, Yikyung Park, Melinda M. Protani, Marina M. Reeves

**Affiliations:** 1 School of Public Health, Faculty of Medicine, The University of Queensland, Brisbane, QLD Australia; 2 Division of Public Health Sciences, Department of Surgery, Washington University School of Medicine, St Louis, MO USA

**Keywords:** Circadian rhythm, Feeding behaviour, Sleep quality, Breast neoplasms, Survival

## Abstract

**Purpose:**

Circadian rhythm disruptors (e.g., night-shift work) are risk factors for breast cancer, however studies on their association with prognosis is limited. A small but growing body of research suggests that altered sleep patterns and eating behaviours are potential mechanistic links between circadian rhythm disruptors and breast cancer. We therefore systematically summarised literature examining the influence of circadian rhythm disrupting behaviours on cancer outcomes in women with breast cancer.

**Methods:**

A systematic search of five databases from inception to January 2021 was conducted. Original research published in English, assessing the relationship between post-diagnosis sleep patters and eating behaviours, and breast cancer outcomes were considered. Risk of bias was assessed using the Newcastle–Ottawa Assessment Scale for Cohort Studies.

**Results:**

Eight studies published original evidence addressing sleep duration and/or quality (*k* = 7) and, eating time and frequency (*k* = 1). Longer sleep duration (≥ 9 h versus [referent range] 6-8 h) was consistently associated with increased risk of all outcomes of interest (HR range: 1.37–2.33). There was limited evidence to suggest that measures of better sleep quality are associated with lower risk of all-cause mortality (HR range: 0.29-0.97). Shorter nightly fasting duration (< 13 h versus ≥ 13 h) was associated with higher risk of all breast cancer outcomes (HR range: 1.21–1.36).

**Conclusion:**

Our review suggests that circadian rhythm disrupting behaviours may influence cancer outcomes in women with breast cancer. While causality remains unclear, to further understand these associations future research directions have been identified. Additional well-designed studies, examining other exposures (e.g., light exposure, temporal eating patterns), biomarkers, and patient-reported outcomes, in diverse populations (e.g., breast cancer subtype-specific, socio-demographic diversity) are warranted.

**Supplementary Information:**

The online version contains supplementary material available at 10.1007/s10549-022-06792-0.

## Introduction

Breast cancer is the most prevalent cancer and the leading cause of cancer-related mortality in women worldwide [1]. Due to advancements in cancer screening and treatments, the number of breast cancer survivors are steadily rising [2]. Despite improvements in longer term prognosis, breast cancer survivors remain at an increased risk of disease progression and mortality [3]. Breast cancer is a heterogeneous disease, with a broad range of factors influencing prognosis [4]. In addition to well-established clinical characteristics [5–7] and demographic factors [8–10], modifiable behaviours such as diet and physical activity have been related to breast cancer outcomes [2]. While research on these established risk factors continue to grow, disruptions to circadian rhythms [11–13] due to night-shift work [14] and light at night [15, 16] have recently been suggested as potential risk factors for breast cancer.

The International Agency for Research on Cancer (IARC) classified circadian-disrupting night-shift work as a probable carcinogen for breast cancer (Group 2A) [17]. Additionally, outdoor light at night that disrupts an individual’s circadian rhythm is also associated with an increased risk of breast cancer [13]. An expert panel review suggested that electric light acted as an effector and enabler of behaviours that may lead to circadian disruption, including inconsistent sleep-wake patterns and night-shift work [18]. Furthermore, late night eating (> 9:30PM) was related to a 48% increased risk of breast cancer (versus ≤ 9:30PM; HR 1.48, 95%CI 1.02–2.17) in the French NutriNet-Santé cohort [19], while another study reported that people who skipped breakfast had a 52% increased risk of cancer-related mortality (HR 1.52, 95%CI 1.06–2.18) compared to those who consumed breakfast regularly [20].

Given this potential role in breast cancer incidence, there is emerging research on the potential mechanistic behaviours linking circadian rhythm disruptors—sleep patterns (e.g., duration, quality, disturbance) and eating behaviours (e.g., frequency of meals, mealtimes)—and disease outcomes following a breast cancer diagnosis. Evidence from the general non-cancer populations suggest, that sleep duration, nightly fasting duration, and timing of eating, are associated with metabolic (glucose, insulin) and inflammatory (C-Reactive Protein) markers [21–24]—pathways that have been implicated in breast cancer progression [25], further supporting the need to examine these behaviours in breast cancer survivors.

Sleep duration is the most commonly studied circadian rhythm disrupting behaviour in relation to cancer survivorship. A recent meta-analysis of sleep duration and cancer mortality in all cancer survivors reported that long sleep duration (≥ 9 h versus referent [ranging from 5–8 h]) in studies assessing either pre- or post-diagnosis was not associated with breast cancer mortality (RR = 1.11, 95%CI 0.74–1.67) [26]. However, strong associations were evident in the small number of studies assessing post-diagnosis long sleep duration (≥ 9 h) only with breast cancer specific mortality (RR = 1.49, 95%CI 1.18–1.89) and all-cause mortality (RR = 1.38, 95%CI 1.16–1.64) [26]. This finding that the effect of sleep duration differs by timing of exposure assessment, suggests that pre- and post- diagnosis measures of behaviours reflective of circadian rhythm disruption need to be considered separately. Nevertheless, this meta-analysis was limited to sleep duration and mortality and did not review studies of sleep quality or other cancer outcomes such as recurrence and progression-free survival. To the best of our knowledge, no study has comprehensively summarised available evidence on the effects of post-diagnosis behaviours that underpin circadian rhythm disruptors on cancer outcomes in breast cancer survivors. Therefore, this systematic review aimed to examine the associations between the behaviours underpinning circadian rhythm disruption and breast cancer outcomes.

## Methods

In accordance with the Preferred Reporting Items for Systematic Reviews and Meta-Analyses (PRISMA) guidelines [27], a systematic search of CINAHL, Embase, MEDLINE, PubMed, and Web of Science from inception to 13 January 2021 was conducted. The search strategy was developed in collaboration with a specialist librarian following the PICOS (Population—Intervention/Exposure—Comparator—Outcome—Study) format and included key terms for breast cancer, prognosis, and circadian disrupting behaviours (see Online Resource 1). For the purpose of this review, circadian rhythm disruption is characterised by behavioural misalignment, defined as the misalignment of eating/fasting cycle, or sleep and wake, with the endogenous central clock [14]. No publication date or language restrictions were applied. Key author searches and manual searches of retrieved full-text articles were conducted for additional publications.

### Study selection

Using Covidence Systematic Review software (Veritas Health Innovation, Melbourne, Australia) [28], duplicates were excluded, and articles were screened against a pre-determined eligibility criteria. Eligible studies (see Online Resource 2) included original research published in English, assessing the relationship between post-diagnosis behaviours reflective of circadian rhythm disruption (e.g., sleep duration, sleep quality, night-time fasting) and breast cancer outcomes (i.e., all-cause mortality, breast cancer-related mortality, breast cancer recurrence, progression-free survival). Study titles, abstracts, and full texts were screened independently by two reviewers. Conflicts were resolved through discussion, and a final decision was made through a process of deliberation and consensus.

### Data extraction and evaluation of study quality

Data from eligible studies were extracted into a formatted database independently by one author (K.D.) and reviewed by three co-authors (M.M.R., Y.P., M.M.P.) to ensure accuracy. Data extracted included study population characteristics, exposure of interest, ascertainment of exposure (mode, timing, and method), outcome(s) assessed, associations, and covariates adjusted in models. The quality of each study was appraised against the Newcastle–Ottawa Assessment Scale for Cohort Studies [29]. Studies were scored ‘Yes (Y)’, ‘Probably Yes (PY)’, ‘No (N)’, ‘Probably No (PN)’, ‘No Information (NI)’ against each criterion to inform risk of bias, and considered as either good, fair, and poor [30]. Due to the heterogeneity in exposures and outcomes assessed, meta-analysis could not be performed. Therefore, a critical assessment of eligible studies was conducted descriptively.

## Results

### Characteristics of included studies

**Fig. 1:**
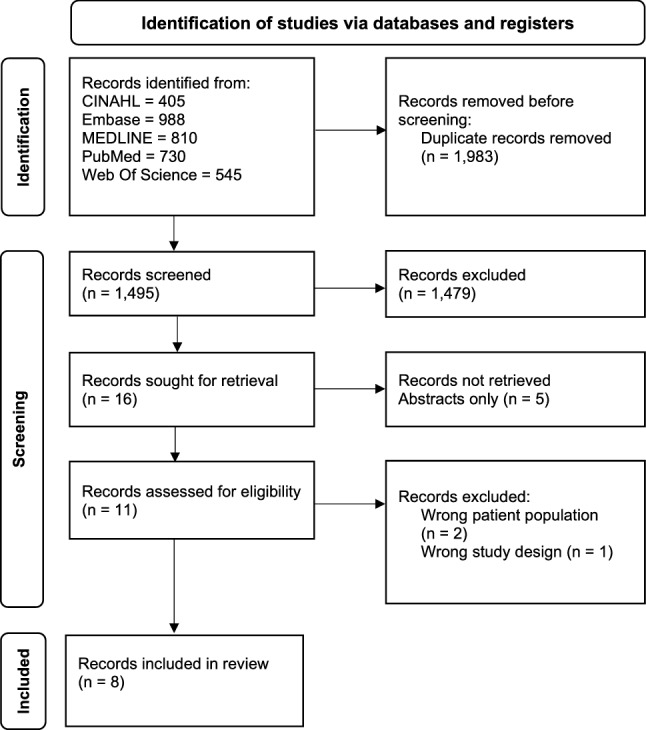
PRISMA flow diagram of included studies [27]

A total of 3,476 records were retrieved across the five databases, of which 1981 duplicates were removed. From the remaining articles (*k* [number of studies] = 1495), 1,479 were excluded based on title and abstract, and eight were excluded based on full-text review. Ultimately, eight studies met the eligibility criteria and were included in this review (Fig. 1). Sleep duration [31–33], sleep quality [32, 33], sleep disorders [34], and eating behaviours [35] were examined in relation to all-cause and breast cancer mortality. The associations of sleep duration and eating behaviours with breast cancer recurrence [31, 35] and sleep duration and sleep quality with progression-free survival [36–38] were reported in these studies.

Characteristics of the studies included are detailed in Online Resource 3. The majority of studies were conducted in the United States (*k* = 5) [31–33, 35, 38] with the remaining studies in the United Kingdom [34], China [37], and Brazil [36]. All eight studies were observational in design. Sample size varied greatly between studies, ranging from 85 [38] to 6,566 [34] (Median: *n* = 1996) breast cancer survivors. Mean age ranged from 46 to 65 years, with four studies recruiting predominantly post-menopausal women [31, 32, 35, 38]. The majority of studies followed-up women ≥ 5 years post breast cancer diagnosis (*k* = 6) [31–35, 38].

### Risk of bias

The risk of bias assessment of included studies is presented in Table 1. Overall, the quality of study methodology and reporting were heterogeneous. With respect to sampling, studies received a lower quality score for selecting cohorts with particular clinical or demographic characteristics, not representative of the overall population of interest [33, 36, 38]. Methodological bias was present in studies that assessed exposures of interest via validated self-reported instruments [31, 32, 35–38], with only two studies receiving high-quality scores for assessing exposures via objective sleep data (actigraphy)[33] and clinician diagnosed sleep disorders [34]. Comparability bias was identified for one study with inadequate assessment for potential confounders [36]. Overall, all studies were allocated high-quality rating with respect to outcome assessment, with only one study receiving a low quality score for insufficient follow-up time [36].

**Table 1:** Quality assessment of included studies using ‘Newcastle–Ottawa Quality Assessment Form for Cohort Studies’ [29]

First author, year of publication, country, study name [ref]	Selection^a^				Comparability^a^	Outcome^a^		
	1	2	3	4^b^	1^c^	1	2^d^	3
Sleep patterns (*k* = 7)								
Bach, 2020, UK [34]	PY	Y	Y	PN	Y	Y	Y	Y
Liang, 2019, China, Guangzhou Breast Cancer Study [37]	PY	Y	PY;PN	PN	Y	PY	PY	Y
Mansano-Schlosser, 2017, Brazil [36]	PN	Y	PY	PN	PN	Y	N	Y
Marinac, 2017, USA, WHEL [31]	PY	Y	PN	PN	Y	Y	Y	Y
Trudel-Fitzgerald, 2017, USA, NHS [32]	PY	Y	PN	PN	Y	Y	Y	Y
Hahm, 2014, USA [38]	PN	Y	PY	N	Y	Y	Y	Y
Palesh, 2014, USA [33]	PN	Y	Y	N	Y	Y	Y	Y
Eating behaviours (*k* = 1)								
Marinac, 2016, USA, WHEL [35]	PY	Y	PY	PN	Y	Y	Y	Y

^a^Selection (1. Representativeness of the exposed cohort, 2. Selection of the non-exposed cohort, 3. Ascertainment of exposure, 4. Demonstration that outcome of interest was not present at start of study); Comparability (1. Comparability of cohorts based on the design or analysis controlled for confounders); Outcomes (1. Assessment of outcome, 2. Was follow-up long enough for outcomes to occur, 3. Adequacy of follow-up of cohorts)

^b^The criterion for absence of outcome at the start of the study was considered met if the cohort excluded those with stage IV/metastatic disease

^c^Age or menopausal status, breast cancer stage or grade, and breast cancer treatment were identified as the key confounders to be considered in analysis

### Sleep patterns

Seven of the eight studies evaluated the effects of sleep patterns on breast cancer outcomes with the majority reporting on sleep duration (*k* = 5) [31–33, 36, 37] followed by sleep quality (*k* = 4) [32, 33, 36, 37]. Sleep disorders [34], habitual daytime napping [37], and bedtime misalignment [38] were also examined (Table 2). Five of seven studies examining sleep-related exposures, ascertained sleep characteristics subjectively through self-reported data collected either via a single-item question (*k* = 2) [31, 37] or validated questionnaires (*k* = 3) [32, 36, 37]. One study used a combination of sleep logs and objectively measured wrist actigraphy data [33] and the other, a clinical diagnosis from a health database [34].

**Table 2:** Associations between post-diagnosis sleep duration, sleep quality, sleep patterns, and eating behaviours and cancer outcomes in breast cancer survivors^a^

	All-cause mortality		Breast cancer mortality		Breast cancer recurrence		Progression-free survival	
	Exposure: HR (95%CI)	Refernces	Exposure: HR (95%CI)	References	Exposure: HR (95%CI)	References	Exposure: HR (95%CI)	References
Sleep duration	≤ 6 h: 0.95 (0.80–1.13)	[31]	≤ 6 h: 0.83 (0.67–1.04)	[31]	≤ 6 h: 0.93 (0.77–1.12)	[31]	< 6 h: 1.45 (0.83–2.54)	[37]
	≤ 6 h: 1.05 (0.88–1.24)	[32]	≤ 6 h: 1.13 (0.86–1.48)	[32]				
	≤ 6 h consistent: 0.91 (0.73–1.12)	[31]	≤ 6 h consistent: 0.78 (0.60–1.01)	[31]	≤ 6 h consistent: 0.93 (0.74 -1.17)	[31]		
	≤ 6 h inconsistent: 0.79 (0.63–0.98)	[31]	≤ 6 h inconsistent: 0.80 (0.61–1.06)	[31]	≤ 6 h inconsistent: 0.82 (0.64–1.06)	[31]		
	≥ 9 h: 1.43 (1.07–1.92)	[31]	≥ 9 h: 1.52 (1.09–2.13)	[31]	≥ 9 h: 1.48 (1.01–2.00)	[31]	> 9 h: 2.33 (1.01–5.42)	[37]
	≥ 9 h: 1.37 (1.10–1.71)	[32]	≥ 9 h: 1.46 (1.02–2.07)	[32]				
	≥ 9 h consistent: 1.46 (0.97–2.21)	[31]	≥ 9 h consistent: 1.22 (0.75–1.99)	[31]	≥ 9 h consistent:1.30 (0.85–1.98)	[31]		
	≥ 9 h inconsistent: 1.47 (1.12–1.93)	[31]	≥ 9 h inconsistent: 1.70 (1.23–2.36)	[31]	≥ 9 h inconsistent: 1.60 (1.18–2.18)	[31]		
	TIB shorter: 0.99 (0.97–1.00)	[33]						
	≥ 1 h increase (from pre-to post- dx)^b^: 1.35 (1.04–1.74)	[32]	≥ 1 h increase (from pre-to post- dx)^b^: 1.29 (0.84–2.00)	[32]				
	≥ 1 h decrease (from pre-to post- dx)^b^: 1.26 (0.97–1.65)	[32]	≥ 1 h decrease (from pre-to post- dx)^b^: 0.89 (0.55–1.45)					
This section should be white							< 6 h or > 9 h: 2.73 (0.99–7.52)^c^	[36]
Sleep quality	Difficulty: 1.49 (1.02–2.19)	[32]	Difficulty: 1.78 (0.94–3.36)	[32]				
	Efficiency (≥ 85%): 0.94 (0.91–0.97)	[33]					Efficiency (< 85%): 1.65 (0.92–2.92)	[37]
							Quality (bad/very bad): 3.08 (1.74–5.47)	[37]
							Quality poor: 0.78 (0.27–2.29)^c^	[36]
	Latency: 0.89 (0.75–1.06)	[33]						
	WASO (min): 0.41 (0.25 -0.67)	[33]						
	WASO (%TST): 0.97 (0.96–0.98)	[33]						
	WE (fewer): 0.93 (0.88 -0.98)	[33]						
	WE duration (shorter): 0.29 (0.14–0.58)	[33]						
Sleep disorders	1.39 (1.04–1.87)	[34]						
Habitual day time napping							0.94 (0.61–1.44)	[37]
Bedtime misalignment ^d^							3.18 (1.32–7.61)	[38]
Nightly fasting	< 13 h: 1.22 (0.95–1.56)	[35]	< 13 h: 1.21 (0.91–1.60)	[35]	< 13 h: 1.36 (1.05–1.76)	[35]		
Eating episodes per day	0.99 (0.89–1.10)	[35]	1.00 (0.89–1.13)	[35]	0.97 (0.87–1.08)	[35]		
Eating after 8PM	Yes: 0.97 (0.76–1.24)	[35]	Yes: 0.98 (0.74–1.28)	[35]	Yes: 0.97 (0.76–1.24)	[35]		

^a^*TIB* Time In Bed; *WASO* Wake After Sleep Onset; *TST* Total Sleep Time; *WE* Wake Episodes

^b^Changes in sleep duration from pre-post-diagnosis by either ⩾1 h more (increase) or ⩾1 h less (decrease), versus no change

^c^Measured relative risk

^d^Defined as misalignment of preferred (assessed via Morningness-Eveningness Questionnaire) and habitual bedtime [38]

Studies that examined the associations with sleep duration, reported increased risk consistently across all outcomes (HR range: 1.37–2.33) for long sleep duration (≥ 9 h versus [referent range] 6–8 h), but not short (< 6 h) [31, 32, 37]. Additionally, in the single study that examined time-varying sleep duration, both consistently and inconsistently sleeping longer (versus consistently sleeping 7-8 h) was associated with all outcomes of interest, however, stronger and statistically significant associations were observed for inconsistently sleeping longer [31]. Poor sleep quality [36, 37] including difficulty (falling asleep or staying asleep) [32], were inconsistently associated with reported outcomes (i.e., all-cause mortality, breast cancer mortality, progression-free survival). There was also limited evidence to suggest that sleep efficiency (≥ 85%), less time awake after sleep onset, and shorter/fewer wake episodes [33] were associated with lower risk, and sleep disorders [34] were associated with increased risk of all-cause mortality.

### Eating behaviours

Only one study assessed eating patterns, evaluating the effects of nightly fasting duration, eating episodes per day, and eating after 8PM on breast cancer outcomes (Table 2) [35]. Eating behaviours were estimated using time stamped dietary intake collected via multiple 24-h dietary recalls collected by phone [35]. A small but consistent increased risk was observed with shorter night-time (< 13 h) fasting across all outcomes (HR range: 1.21–1.36) [35]. Associations for eating occasions and eating after 8 pm were suggestive of no association with any of the outcomes [35].

## Discussion

This review provides a comprehensive summary on the limited evidence available on post-diagnosis behaviours that underpin circadian rhythm disruptors and breast cancer outcomes. Our updated search on sleep duration and breast cancer survival did not retrieve any additional studies and found that longer sleep duration (≥ 9 h) was associated with increased risk of breast cancer outcomes, which is consistent with the results of the meta-analysis by Stone and colleagues [26]. Similarly, among the general population, long sleep duration (> 8 h versus 6-8 h) has been associated with increased risk of mortality, incident diabetes mellitus, cardiovascular disease, and obesity [39].

Findings from our systematic review also suggest that sleep quality including sleep difficulty is associated with all-cause mortality and possibly poor progression-free survival. Gottfried and colleagues reported similar results whereby, poor sleep quality (i.e., frequent arousals at night) determined via a self-reported questionnaire (standard practice on ward admission) was highly related to overall survival amongst lung cancer survivors (versus no sleep problems: HR 2.04, 95%CI 1.37–3.05) [40]. Chronically poor sleep is also associated with patient-reported outcomes and poor cancer prognosis such as fatigue, poor quality of life, impaired immune function, and development of comorbidities [32, 33].

Of importance to note, the observational design of the included studies makes them susceptible to reverse causation and potential confounding biases. It is currently unclear whether long sleep duration and disrupted sleep are caused by progressing or recurrent underlying disease, rather than sleep behaviour directly affecting cancer progression. In the studies that examined sleep duration across clinical cancer characteristics at diagnosis, those with more advanced disease (stage III) were only slightly more likely to report longer sleep duration (≥ 9 h) [31, 32], and all studies reporting detrimental associations with long sleep duration were independent of tumour stage at diagnosis—a measure of disease severity associated with recurrence risk [31, 32, 37].

Poor sleep quality can be explained in part by the side-effects of cancer treatment, including the occurrence or exacerbation of menopausal symptoms (e.g., hot flashes) and fatigue [41, 42]. Poor sleep quality and sleep disturbance remain prevalent problems years after cancer treatment completion [43]. All but two studies [34, 36] examining sleep duration and sleep quality, controlled for cancer treatments (receipt of chemotherapy, radiotherapy, hormone therapy) in their analyses [31–33, 37, 38], reporting that long sleep duration and poor sleep quality were associated with cancer outcomes in women with a breast cancer diagnosis, independent of the type of treatment received. However, none of the included studies adjusted for side-effects like fatigue, commonly experienced during chemotherapy and radiation treatment [44]. Additionally, in the studies included in this review, the timing of collection of sleep data with respect to commencement of cancer treatment varied, ranging from pre-chemotherapy treatment [38] to seven years post diagnosis [32]. Despite attempts to examine independent associations of these behaviours, the included observational studies are likely to have residual confounding by unmeasured confounders or imprecisely measured confounders (e.g., fatigue, depression, physical activity), which may attenuate or overestimate the strength of an association.

In the single study examining meal timing, a statistically significant association was observed between short nightly fasting duration (< 13 h) and breast cancer recurrence, with small suggestive associations for all-cause and breast cancer mortality [35]. Although light is the most dominant zeitgeber (German for ‘timekeeper’) for the master clock in the suprachiasmatic nucleus, which maintains circadian rhythms, eating and activity schedules play a key role in entraining peripheral clocks located throughout our body, including all organs [45]. The potential role of eating behaviours in breast cancer progression is supported by research on the association of time restricted eating (a dietary approach in which all caloric intake is consolidated into a set period during the active phase of the day, without necessarily altering diet patterns) [46] and biomarkers implicated in breast cancer. For example, in adult women without a history of breast cancer and diabetes, time restricted eating regimens were associated with improved glucoregulation [47], lower glycated haemoglobin (HbA_1c_) levels [24], and lower C-reactive protein (CRP) levels [23]. Similar influence of eating behaviours reflective of circadian rhythm disruption are observed in other hormonal cancers. For example, in a sample of adults without a cancer diagnosis at baseline, timing of the last eating episode (> 9:30PM versus ≤ 9:30PM) was associated with increased risk of prostate cancer [19].

Our systematic review is not without limitations, inherent to the design of included studies. The results of this study are based on predominantly white, postmenopausal breast cancer survivors, limiting generalizability to ethnically diverse and younger breast cancer survivors. Additionally, all eligible studies are observational and predominantly retrospective in nature, whereby causality is not made clear. Furthermore, although most studies measured exposures using validated questionnaires and single-item questions, self-reported responses are subjective and less accurate than those using objective measurements (e.g., activity monitors) – but studies using objective measurements of these behaviours could still be prone to reverse causality bias. Lastly, the small number of significantly heterogeneous eligible studies limited our ability to conduct a meta-analysis to estimate the pooled effect across included studies.

Our review, although narrative, comprehensively synthesizes the association between post-diagnosis behaviours reflective of circadian rhythm disruption and breast cancer outcomes, and has identified opportunities for future research. Available evidence has largely viewed women with breast cancer as a homogenous population. However, breast cancer is a heterogeneous disease characterized by different cell types and molecular subtypes [4]. Therefore, future studies need to explore the associations of circadian-disrupting behaviours and cancer outcomes by breast cancer subtypes and in more diverse breast cancer populations (including race/ethnic, age, and socio-demographical diversity), which will inform recommendations for breast cancer survivors. Additionally, while studies to date have primarily focused on sleep patterns and breast cancer outcomes, further studies are needed that examine other circadian rhythm disrupting behaviours such as light at night, and temporal eating patterns on breast cancer outcomes. Furthermore, while possible, the literature is yet to fully examine [35] the joint effects and mutually independent associations of sleep patterns and eating behaviours on breast cancer outcomes. This is of particular interest given the significant positive association between sleep duration and nightly fasting duration [35]. Future studies therefore need to consider both the independent and joint effects of circadian rhythm disrupting behaviours on breast cancer outcomes. In addition to understanding associations with hard cancer endpoints, an understanding of the association between circadian-disrupting behaviours and biomarkers implicated in breast cancer progression as well as patient-reported outcomes such as treatment-related side-effects like fatigue, will provide a deeper understanding of the biological mechanisms involved in these associations. Longitudinal studies collecting repeated measures of both circadian-disrupting behaviours as well as early cancer progression biomarkers may also help to elucidate the direction of causality between circadian rhythm and cancer progression. Other methodological approaches, such as mendelian randomisation and Directed Acyclic Graphs (DAGs), may also be useful in helping to establish temporality and appropriately control for confounding. Several single nucleotide polymorphisms (SNPs) have been associated with chronotype, sleep duration and insomnia, and have been shown to be associated with breast cancer risk, however no such SNPs exist for eating patterns [48]. Future studies should also consider the use of DAGs to ensure that all key variables are measured and appropriately accounted for in analyses to minimise residual confounding.

As the number of breast cancer survivors continue to rise worldwide, the implications of circadian rhythm disrupting behaviours on breast cancer outcomes are of growing interest. This review summarized the limited literature available, suggesting post-diagnosis sleep patterns and eating behaviours reflective of circadian rhythm disruption may have adverse effects on cancer outcomes in breast cancer survivors.

## Supplementary Information

Below is the link to the electronic supplementary material.

Supplementary file1 (PDF 129 KB)

## Data Availability

All data are incorporated into the article and its online supplementary material.

## References

[1] Sung H, Ferlay J, Siegel RL, Laversanne M, Soerjomataram I, Jemal A (2021). Global cancer statistics 2020: Globocan estimates of incidence and mortality worldwide for 36 cancers in 185 countries. CA: A Cancer J for Clinic.

[2] World Cancer Research Fund International/American Institute for Cancer Research. Continuous update project report: Diet, nutrition, physical activity, and breast cancer survivors 2014 https://www.wcrf.org/wp-content/uploads/2021/03/Breast-Cancer-Survivors-2014-Report.pdf. Accessed 13 Nov 2021

[3] Levi F, Bosetti C, Lucchini F, Negri E, La Vecchia C (2005). Monitoring the decrease in breast cancer mortality in europe. Eur J Cancer Prev.

[4] Polyak K (2011). Heterogeneity in breast cancer. J Clin Invest.

[5] Nicolini A, Giardino R, Carpi A, Ferrari P, Anselmi L, Colosimo S (2006). Metastatic breast cancer: An updating. Biomed Pharmacother.

[6] Schwartz AM, Henson DE, Chen D, Rajamarthandan S (2014). Histologic grade remains a prognostic factor for breast cancer regardless of the number of positive lymph nodes and tumor size: A study of 161 708 cases of breast cancer from the seer program. Arch Pathol Lab Med.

[7] Solak M, Turkoz FP, Keskin O, Aksoy S, Babacan T, Sarici F (2015). The lymph node ratio as an independent prognostic factor for non-metastatic node-positive breast cancer recurrence and mortality. J buon.

[8] Chen HL, Zhou MQ, Tian W, Meng KX, He HF (2016). Effect of age on breast cancer patient prognoses: A population-based study using the seer 18 database. PLoS ONE.

[9] Chlebowski RT, Chen Z, Anderson GL, Rohan T, Aragaki A, Lane D (2005). Ethnicity and breast cancer: Factors influencing differences in incidence and outcome. J Natl Cancer Inst.

[10] Arce-Salinas C, Aguilar-Ponce JL, Villarreal-Garza C, Lara-Medina FU, Olvera-Caraza D, Alvarado Miranda A (2014). Overweight and obesity as poor prognostic factors in locally advanced breast cancer patients. Breast Cancer Res Treat.

[11] Manouchehri E, Taghipour A, Ghavami V, Ebadi A, Homaei F, Latifnejad Roudsari R (2021). Night-shift work duration and breast cancer risk: An updated systematic review and meta-analysis. BMC Womens Health.

[12] Xiao Q, James P, Breheny P, Jia P, Park Y, Zhang D (2020). Outdoor light at night and postmenopausal breast cancer risk in the nih-aarp diet and health study. Int J Cancer.

[13] Lai KY, Sarkar C, Ni MY, Cheung LWT, Gallacher J, Webster C (2021). Exposure to light at night (lan) and risk of breast cancer: A systematic review and meta-analysis. Sci Total Environ.

[14] Qian J, Scheer F (2016). Circadian system and glucose metabolism: Implications for physiology and disease. Trends Endocrinol Metab.

[15] Czeisler CA, Duffy JF, Shanahan TL, Brown EN, Mitchell JF, Rimmer DW (1999). Stability, precision, and near-24-hour period of the human circadian pacemaker. Science.

[16] Roenneberg T, Merrow M (2016). The circadian clock and human health. Curr Biol.

[17] International Agency for Research on Cancer. Iarc monographs volume 124: Night shift work 2019 https://www.iarc.who.int/news-events/iarc-monographs-volume-124-night-shift-work/. Accessed 26 Nov 2021

[18] Lunn RM, Blask DE, Coogan AN, Figueiro MG, Gorman MR, Hall JE (2017). Health consequences of electric lighting practices in the modern world: A report on the national toxicology program's workshop on shift work at night, artificial light at night, and circadian disruption. Sci Total Environ.

[19] Srour B, Plancoulaine S, Andreeva VA, Fassier P, Julia C, Galan P (2018). Circadian nutritional behaviours and cancer risk: New insights from the nutrinet-santé prospective cohort study: Disclaimers. Int J Cancer.

[20] Helo D, Appiah L, Bhende KM, Byrd TL, Appiah D (2021). The association of skipping breakfast with cancer-related and all-cause mortality in a national cohort of united states adults. Cancer Causes Control.

[21] Mesarwi O, Polak J, Jun J, Polotsky VY (2013). Sleep disorders and the development of insulin resistance and obesity. Endocrinol Metab Clin North Am.

[22] Chiang J-K (2014). Short duration of sleep is associated with elevated high-sensitivity c-reactive protein level in taiwanese adults: A cross-sectional study. J Clin Sleep Med.

[23] Marinac CR, Sears DD, Natarajan L, Gallo LC, Breen CI, Patterson RE (2015). Frequency and circadian timing of eating may influence biomarkers of inflammation and insulin resistance associated with breast cancer risk. PLoS ONE.

[24] Marinac CR, Natarajan L, Sears DD, Gallo LC, Hartman SJ, Arredondo E (2015). Prolonged nightly fasting and breast cancer risk: Findings from nhanes (2009–2010). Cancer Epidemiol Biomarkers Prev.

[25] Brenner DR, Brockton NT, Kotsopoulos J, Cotterchio M, Boucher BA, Courneya KS (2016). Breast cancer survival among young women: A review of the role of modifiable lifestyle factors. Cancer Causes Control.

[26] Stone CR, Haig TR, Fiest KM, McNeil J, Brenner DR, Friedenreich CM (2019). The association between sleep duration and cancer-specific mortality: A systematic review and meta-analysis. Cancer Causes Control.

[27] Page MJ, McKenzie JE, Bossuyt PM, Boutron I, Hoffmann TC, Mulrow CD (2021). The prisma 2020 statement: An updated guideline for reporting systematic reviews. BMJ.

[28] Covidence systematic review software: Veritas Health Innovation; www.covidence.org. Accessed 13 Jan 2021

[29] GA Wells BS, D O'Connell, J Peterson, V Welch, M Losos, P Tugwell. The newcastle-ottawa scale (nos) for assessing the quality of nonrandomised studies in meta-analyses 2013 http://www.ohri.ca/programs/clinical_epidemiology/oxford.asp. Accessed 06 Dec 2021

[30] Thresholds for converting the newcastle-ottawa scales to ahrq standards (good, fair, and poor) https://www.ncbi.nlm.nih.gov/books/NBK115843/bin/appe-fm3.pdf. Accessed 06 Dec 2021

[31] Marinac CR, Nelson SH, Flatt SW, Natarajan L, Pierce JP, Patterson RE (2017). Sleep duration and breast cancer prognosis: Perspectives from the women's healthy eating and living study. Breast Cancer Res Treat.

[32] Trudel-Fitzgerald C, Zhou ES, Poole EM, Zhang X, Michels KB, Eliassen AH (2017). Sleep and survival among women with breast cancer: 30 years of follow-up within the nurses' health study. Br J Cancer.

[33] Palesh O, Aldridge-Gerry A, Zeitzer JM, Koopman C, Neri E, Giese-Davis J (2014). Actigraphy-measured sleep disruption as a predictor of survival among women with advanced breast cancer. Sleep.

[34] Bach L, Kalder M, Kostev K (2021). Depression and sleep disorders are associated with early mortality in women with breast cancer in the united kingdom. J Psychiatr Res.

[35] Marinac CR, Nelson SH, Breen CI, Hartman SJ, Natarajan L, Pierce JP (2016). Prolonged nightly fasting and breast cancer prognosis. JAMA Oncol.

[36] Mansano-Schlosser TC, Ceolim MF (2017). Association between poor clinical prognosis and sleep duration among breast cancer patients. Rev Lat Am Enfermagem.

[37] Liang ZZ, Zhang YX, Lin Y, Liu Q, Xie XM, Tang LY (2019). Joint effects of multiple sleep characteristics on breast cancer progression by menopausal status. Sleep Med.

[38] Hahm BJ, Jo B, Dhabhar FS, Palesh O, Aldridge-Gerry A, Bajestan SN (2014). Bedtime misalignment and progression of breast cancer. Chronobiol Int.

[39] Jike M, Itani O, Watanabe N, Buysse DJ, Kaneita Y (2018). Long sleep duration and health outcomes: A systematic review, meta-analysis and meta-regression. Sleep Med Rev.

[40] Gottfried T, Kamer I, Salant I, Urban D, Lawrence YR, Onn A (2020). Self-reported sleep quality as prognostic for survival in lung cancer patients. Cancer Manag Res.

[41] Savard J, Davidson JR, Ivers H, Quesnel C, Rioux D, Dupéré V (2004). The association between nocturnal hot flashes and sleep in breast cancer survivors. J Pain Sympt Manage.

[42] Liu L, Rissling M, Natarajan L, Fiorentino L, Mills PJ, Dimsdale JE (2012). The longitudinal relationship between fatigue and sleep in breast cancer patients undergoing chemotherapy. Sleep.

[43] Strollo SE, Fallon EA, Gapstur SM, Smith TG (2020). Cancer-related problems, sleep quality, and sleep disturbance among long-term cancer survivors at 9-years post diagnosis. Sleep Med.

[44] Liu L, Mills PJ, Rissling M, Fiorentino L, Natarajan L, Dimsdale JE (2012). Fatigue and sleep quality are associated with changes in inflammatory markers in breast cancer patients undergoing chemotherapy. Brain Behav Immun.

[45] Buttgereit F, Smolen JS, Coogan AN, Cajochen C (2020). Time-restricted eating: Benefits, mechanisms, and challenges in translation. iScience.

[46] Regmi P, Heilbronn LK (2020). Time-restricted eating: Benefits, mechanisms, and challenges in translation. iScience.

[47] Patterson RE, Laughlin GA, LaCroix AZ, Hartman SJ, Natarajan L, Senger CM (2015). Intermittent fasting and human metabolic health. J Acad Nutr Diet.

[48] Richmond RC, Anderson EL, Dashti HS, Jones SE, Lane JM, Strand LB (2019). Investigating causal relations between sleep traits and risk of breast cancer in women: Mendelian randomisation study. BMJ.

