# Supplementary material for: Circadian rhythm disrupting behaviours and cancer outcomes in breast cancer survivors: a systematic review
Source: Breast Cancer Res Treat. 2022 Nov 23;198(3):413–21. doi: 10.1007/s10549-022-06792-0 (PMC10036454; doi:10.1007/s10549-022-06792-0)
Supplement: Supplementary file 1 — Supplementary file1 (PDF 129 KB) [file 10549_2022_6792_MOESM1_ESM.pdf]

Circadian rhythm disrupting behaviours and cancer outcomes in breast cancer survivors: A systematic review

Breast Cancer Research and Treatment

Kelly D'cunha, MDiet[1]
Yikyung Park, ScD[2]
Melinda M. Protani, PhD[1]
Marina M. Reeves, PhD[1]

1. Faculty of Medicine, School of Public Health, The University of Queensland, Brisbane, QLD, Australia
2. Division of Public Health Sciences, Department of Surgery, Washington University School of Medicine, St Louis, MO, United States of America

Correspondence: Kelly D'cunha, Faculty of Medicine, School of Public Health, The University of Queensland, Brisbane, QLD, Australia; k.dcunha@uq.edu.au ; +61 478 846 330

Online Resource 1:

Search strategy applied across title and abstracts on the PubMed database

1. ‘breast cancer*’ OR ‘breast neoplasm*’ OR ‘breast carcinoma’ OR ‘breast tumor’ OR ‘breast tumor’ OR ‘mammary cancer*’ OR ‘mammary neoplasm*’ OR ‘mammary carcinoma’ OR ‘mammary tumour’ OR mammary tumor’
2. prognosis (MeSH) OR mortality OR survival OR death OR recurrence OR progression OR outcome* OR fatal OR prediction OR biomark*
3. circadian

   OR

   ‘eating time’ OR ‘meal time’ OR ‘eating frequency’ OR ‘eating occasion’ OR ‘temporal eating pattern’ OR chrononutrition OR ‘nightly fasting’ OR ‘night eating syndrome’

   OR

   sleep AND (duration OR disturbance OR efficiency OR effective OR quality OR regularity OR latency) OR ‘sleep apnea’ OR ‘sleep apnoea’ OR insomnia OR ‘sleep wake cycle’ OR ‘sleep wake disorders (MeSH)

1. AND 2. AND 3.

Online Resource 2

Table 1: PICOS criteria summary for systematic review.

	Inclusion	Exclusion
Population	Women with a history of breast cancer diagnosis	Women with diagnosed concurrent condition likely to influence short-term survival<br>Participants diagnosed with cancers other than breast cancer
Intervention/Exposure	Sleep/wake cycle including sleep duration, quality, efficiency, regularity, disturbance, and sleep wake disorders<br>Feeding/fasting cycles including mealtimes, meal frequency, eating occasions and nightly fasting	Exposures assessed before cancer diagnosis<br>Dietary intakes, dietary patterns based on intakes, diet quality/quantity<br>Only evaluated circadian phase markers, including melatonin and core body temperature, as well as cortisol and other endocrine hormones.
Comparator	This study does not lend itself to comparators or controls	
Outcomes	Prognosis and prognostic biomarkers including mortality, recurrence, progression, death, survival	(Health related) Quality of life<br>Incidence /Risk
Study	Experimental and observational human studies	Case reports, Case series, Case studies<br>Genetic and cell studies<br>Animal studies<br>Systematic reviews and Meta Analyses<br>Studies not in English<br>Studies for which full text is not available (after contacting author)

Online Resource 3

Table 1: Summary of included studies

First author, Year (Reference), Country, Study name	Sample characteristics	Exposure	Ascertainment of exposure: Mode, timing, method	Outcome	Adjustments
		**Sleep patterns (k = 7)**			
Bach, 2020 (34), UK	Women first diagnosed with breast cancer in one of 200 general practices in the UK between January 2008 and December 2012 (index date).<br><br>n = 6,656<br><br>Mean age: 57.9y (SD 12)<br><br>Post-menopausal: No information<br><br>Ethnicity: No information<br><br>Exclusion:<br>Women with other cancer diagnoses and women with primary metastatic breast cancer prior to the index date.	Sleep disorders	Clinic health database - clinician diagnosed sleep disorders<br><br>Post-diagnosis<br><br>Sleep disorder diagnosis data was obtained from the Disease Analyzer database (IQVIA).	All-cause mortality	Age, metastases in the period 4 months–5 years after the index date, and tamoxifen and aromatase inhibitor therapy<br><br>Diabetes mellitus, obesity, lipid metabolism disorders, atrial hypertension, ischemic heart diseases, renal insufficiency, thyroid gland disorders, depression, sleep disorders
Liang, 2019 (37), China, GBCS	Women with pathologically confirmed primary breast cancer from the First and the Second Affiliated Hospitals and the Cancer Center of Sun Yat-sen University	Sleep duration<br><br>Sleep quality<br><br>Sleep efficiency	Self-reported<br><br>Post-diagnosis<br><br>Sleep duration was ascertained by asking the participants	Progression free survival	Age, menopausal status, cancer stage, chemotherapy, hormone therapy, BMI

First author, Year (Reference), Country, Study name	Sample characteristics	Exposure	Ascertainment of exposure: Mode, timing, method	Outcome	Adjustments
	in Guangzhou between October 2008 and December 2014.<br><br>n = 1,580<br><br>Median age = 46y (IQR 40-55)<br><br>Post-menopausal = 34%<br><br>Ethnicity: No information<br><br>Exclusion:<br>Women who reported disease progression before sleep assessment and carcinoma in situ.	Habitual daytime napping	about their total hours of actual sleep at night.<br><br>Sleep quality was scored using the single sleep quality item of the PSQI.<br><br>Sleep efficiency was calculated as the ratio of actual sleep duration at night to time in bed to present continuity of sleep.<br><br>Daytime napping was defined as napping at least three times a week.		HER2 status, scores of Charlsons Comorbidity Index, education level
Mansano-Schlosser, 2017 (36), Brazil	Women 18 years or older diagnosed with breast cancer from the Oncology Ward and Breast Cancer Clinics of a university hospital in Brazil.<br><br>n = 114<br><br>Mean age: 55.9y (SD 11.7)<br><br>Post-menopausal: No information	Sleep duration<br><br>Sleep quality	Self-reported<br><br>Post-diagnosis<br><br>Sleep duration and quality was ascertained via the Pittsburgh Sleep Quality Index (total score; validated in Brazil).	Poor clinical progression	Cancer stage, tumor size<br><br>Her2 status, estrogen receptor, progesterone receptor, lymphatic invasion

First author, Year (Reference), Country, Study name	Sample characteristics	Exposure	Ascertainment of exposure: Mode, timing, method	Outcome	Adjustments
	Ethnicity: White – 72.8%<br><br>Exclusion:<br>Women with a Karnofsky Scale score lower than 70, inadequate medical or emotional condition to respond to an interview, women with data not recovered from medical records and women who dropped out of the study.				
Marinac, 2017 (31), USA, WHEL 28	Women sampled from the Women's Healthy Eating and Living Study who survived breast cancer.<br><br>n = 3047<br><br>Mean age: 52.8y (SD 9)<br><br>Post-menopausal: 79.4%<br><br>Ethnicity: White, non-Hispanic – 85.2%<br><br>Exclusion:<br>Women with objective evidence of recurrent disease, receiving estrogen replacement therapy,	Sleep duration	Self-reported<br><br>Post-diagnosis<br><br>Sleep duration was ascertained by asking participants "About how many hours of sleep did you get on a typical night during the past 4 weeks?".	All-cause mortality<br><br>Breast cancer-specific mortality<br><br>Breast cancer recurrence	Age, menopausal status, cancer stage, cancer grade, treatment, chemotherapy, radiotherapy, endocrine therapy<br><br>HER2 status, BMI, number of co-morbidities, race/ethnicity, physical activity

First author, Year (Reference), Country, Study name	Sample characteristics	Exposure	Ascertainment of exposure: Mode, timing, method	Outcome	Adjustments
	diagnosis of cirrhosis, diagnosis of other primary or recurrent invasive cancer within 10 years of recruitment date (1995), any previous diagnosis of invasive breast carcinoma, other criteria specific to the dietary intervention.				
Trudel-Fitzgerald, 2017 (32), USA, NHS	Women who reported sleep duration within four years following an invasive breast cancer diagnosis from the Nurses' Health Study.<br><br>n = 3682<br><br>Mean age: 64.9y (SD 9.3)<br><br>Post-menopausal: 88.3%<br><br>Ethnicity: No information<br><br>Exclusion:<br>Women who reported a cancer before their breast cancer diagnosis or stage IV diagnosis.	Sleep duration<br><br>Sleep changes pre- and post-diagnosis<br><br>Sleep difficulty	Self-reported<br><br>Post-diagnosis<br><br>Sleep duration was ascertained by asking participants about 'total hours of actual sleep in a 24-hour period'<br><br>Sleep changes pre and post-diagnosis were assessed by women were categorized as sleeping either ≥1 h less, ≥1 h more, or no change.<br><br>Sleep difficulties were assessed by querying about 'How much of the time during the past 4 weeks did you have difficulty falling asleep or staying asleep?'.	Overall survival	Age, menopausal status, cancer stage, chemotherapy, radiation therapy, surgery, hormone therapy<br><br>Year of diagnosis, time since diagnosis, prevalent diabetes or heart disease, missing indicators for oncologic treatments, marital status, education level, income, oral contraceptive use, number of pregnancies, family history of breast cancer, post-menopausal hormone use, BMI, post-diagnosis sleep duration, alcohol intake, caffeine, energy intake, physical activity

First author, Year (Reference), Country, Study name	Sample characteristics	Exposure	Ascertainment of exposure: Mode, timing, method	Outcome	Adjustments
Hahm, 2014 (38), USA	Women diagnosed with metastatic or recurrent breast cancer who had a Karnofsky performance ratings of at least 70%.<br><br>n = 85<br><br>Mean age: 46.1y (SD 8)<br><br>Post-menopausal: 100%<br><br>Ethnicity: White, non-Hispanic – 88.2%<br><br>Exclusion:<br>Women who had bilateral lymph nodes removed, had other active cancers within the past 10 years, had a history of major psychiatric illness that required hospitalization in the preceding year, exhibited substance abuse or dependence, or engaged in travel involving two or more time zones or shift work during the 2 weeks prior to study commencement.	Bedtime misalignment	Self-reported<br><br>Post-diagnosis<br><br>Chronotype and preferred bedtime was ascertained using Morning-Evening Questionnaire (MEQ).<br><br>Habitual bedtime was assessed through sleep-wake log completed within 30 minutes of arising each morning for two weeks.	Disease free survival	Age, cancer stage, chemotherapy, radiation therapy, hormonal therapy, Herceptin therapy, surgery<br><br>Receptor status at initial diagnosis, treatment, circulating NK cell count, Karnofsky performance rating, severity and interference index of pain, depression, trait and state anxiety, posttraumatic stress disorder rating, marital status, educational level, income, employment status

First author, Year (Reference), Country, Study name	Sample characteristics	Exposure	Ascertainment of exposure: Mode, timing, method	Outcome	Adjustments
Palesh, 2014 (33), USA	Women diagnosed metastatic or locally advanced breast cancer, had Karnofsky performance ratings of at least 70%, residing in the greater San Francisco Bay area<br><br>n = 97<br><br>Mean age: 54.6y (SD 9.8)<br><br>Post-menopausal: No information<br><br>Ethnicity: White, non-Hispanic – 86.6%<br><br>Exclusion:<br>Women with other active cancers within the past 10 years, had positive supraclavicular lymph nodes as the only metastatic lesion, had a diagnosis of a concurrent medical condition likely to influence short-term survival, used corticosteroids within the preceding month, or reported a history of major psychiatric illness requiring hospitalization or current alcohol or drug abuse/dependence.	Sleep duration<br><br>Sleep efficiency<br><br>Sleep latency<br><br>Wake after sleep onset (in minutes)<br><br>Wake after sleep onset (% of Total sleep time)<br><br>Mean number of wake episodes<br><br>Mean weak episode duration	Objective<br><br>Post-diagnosis<br><br>Sleep duration and disruption was ascertained via a wrist actigraph worn for three consecutive days.<br><br>Self-reported<br><br>Post-diagnosis<br><br>Sleep diary was used to document bedtime, time at awakening, and any periods during which the actigraph was removed.	Overall survival	Age, disease status, treatment<br><br>Medication, depression, salivary cortisol; estrogen receptor status, metastatic disease spread

First author, Year (Reference), Country, Study name	Sample characteristics	Exposure	Ascertainment of exposure: Mode, timing, method	Outcome	Adjustments
			**Eating behaviours (k = 1)**		
Marinac, 2016 (35), USA, WHEL	Women diagnosed with early-stage invasive breast cancer from the WHEL study who had time-stamped, 24- hour dietary recall data.<br><br>n = 2413<br><br>Mean age: 52.4y (SD 8.9)<br><br>Post-menopausal: 78.5%<br><br>Ethnicity: White, non-Hispanic – 85.5%<br><br>Exclusion:<br>Women who reported diabetes at baseline.	Nightly fasting	Self-reported<br><br>Post-diagnosis<br><br>Dietary intake was assessed by 24- hour dietary recalls collected via telephone on random days during a 3-week period, stratified for weekend versus weekdays.<br><br>Nightly fasting duration was estimated by calculating the elapsed hours between first and last eating episode, subtracted from 24 hours	All-cause mortality<br><br>Breast cancer mortality<br><br>Breast cancer recurrence	Age, menopausal status, tumor stage, tumor grade, treatment<br><br>Race/ethnicity, education level, number of co-morbidities, daily intake (in kilocalories), eating episodes per day, and eating after 8 PM
